# Expression of excess receptors and negative feedback control of signal pathways are required for rapid activation and prompt cessation of signal transduction

**DOI:** 10.1186/1478-811X-7-3

**Published:** 2009-03-03

**Authors:** Hiroshi Kobayashi, Ryuzo Azuma, Takuo Yasunaga

**Affiliations:** 1 Department of Biochemistry, Graduate School of Pharmaceutical Sciences, Chiba University, 1-8-1, Inohana, Chuo-ku, Chiba 260-8675, Japan; 2 Department of Bioscience and Bioinformatics, Graduate School of Computer Science and Systems Engineering, Kyushu Institute of Technology, 680-4 Kawazu, Iizuka, Fukuoka 820-0067, Japan; 3 Japan Science and Technology Agency, Core Research for Evolutional Science and Technology (JST/CREST), Sanbancho Bldg, 5, Sanbancho, Chiyoda-ku, Tokyo 102-0075, Japan

## Abstract

**Background:**

Cellular signal transduction is initiated by the binding of extracellular ligands to membrane receptors. Receptors are often expressed in excess, and cells are activated when a small number of receptors bind ligands. Intracellular signal proteins are activated at a high level soon after ligand binding, and the activation level decreases in a negative feedback manner without ligand clearance. Why are excess receptors required? What is the physiological significance of the negative feedback regulation?

**Results:**

To answer these questions, we developed a Monte Carlo simulation program to kinetically analyze signal pathways using the model in which ligands are bound to receptors and then membrane complexes with other membrane proteins are formed. Our simulation results showed that excess receptors are not required for cell activation when the dissociation constant (Kd) of the ligand-receptor complex is 10^-10 ^M or less. However, such low Kd values cause delayed signal shutdown after ligand clearance from the extracellular space. In contrast, when the Kd was 10^-8 ^M and the ligand level was less than 1 μM, excess receptors were required for prompt signal propagation and rapid signal cessation after ligand clearance. An initial increase in active cytosolic signal proteins to a high level is required for rapid activation of cellular signal pathways, and a low level of active signal proteins is essential for the rapid shutdown of signal pathways after ligand clearance.

**Conclusion:**

The present kinetic analysis revealed that excess receptors and negative feedback regulation promote activation and cessation of signal transduction with a low amount of extracellular ligand.

## Background

Cellular signal transduction is mediated by a complex system involving many proteins. Experimental studies have unraveled several transduction pathways and the roles of many proteins, but many questions remain unanswered. It has been shown that successful cellular activation only requires ligand binding by a small fraction of the available receptors. For example, HeLa cells have been shown to express approximately 50,000 EGF receptors per cell, but binding of only 300 EGF molecules to the cell surface is sufficient to activate 50% of cells [1]. Why do cells express an excess number of receptors?

It is generally accepted that the binding of extracellular ligands to membrane receptors initiates the phosphorylation of signal proteins in a stepwise manner. Many studies have suggested that the majority of signal proteins are phosphorylated immediately after the binding of a ligand to its receptor, after which the level of signal protein decreases [2-7]. To explain this change, it has been hypothesized that the inactivation of signal proteins is regulated in a negative feedback manner by the active form of the signal protein of a late reaction step, thereby decreasing the levels of active proteins [8-10]. However, it remains unclear why this kind of negative feedback regulation is required.

Kinetic analysis is a useful way to analyze these questions. Simple reactions that are mediated by a single enzyme have been analyzed using classical enzyme kinetics, but this method is hard to apply to the kinetic analysis of signal pathways because of their complexity. Recently, an elegant way to facilitate quantification has been developed with the aid of computers. Two types of computer simulation techniques are now available for theoretical studies of biological phenomena: numerical integration of differential equations and Monte Carlo simulation. The former method can be used to evaluate average behavior involving a large number of molecules and stochastic variation. In contrast, the latter can simulate both population behavior and single molecule dynamics. Monte Carlo simulation can also address time-dependent fluctuations involving noise as well as cell-to-cell population heterogeneity.

Receptor-ligand complex formation has been simulated using Monte Carlo techniques [11-13], but these previous analyses have not answered the above questions. Our group developed Monte Carlo simulation programs using a conventional personal computer with Windows XP or 2000 operating systems to examine the kinetic significance of the clustering of membrane receptors and their associate proteins and found that the pre-clustering of such proteins promotes cellular signaling [14]. In the present study, our previous technique was applied to clarify why cells have an excess amount of receptors and why negative feedback regulation is required. We used the following model in the present simulation. Extracellular ligands are bound to receptors and then membrane complexes with other membrane proteins are formed. In this model, pre-clustering of membrane proteins is required for efficient cell activation as shown previously [14]. The complex activates the first cytosolic signal protein and other signal proteins are activated step by step.

## Methods

**Figure 1:**
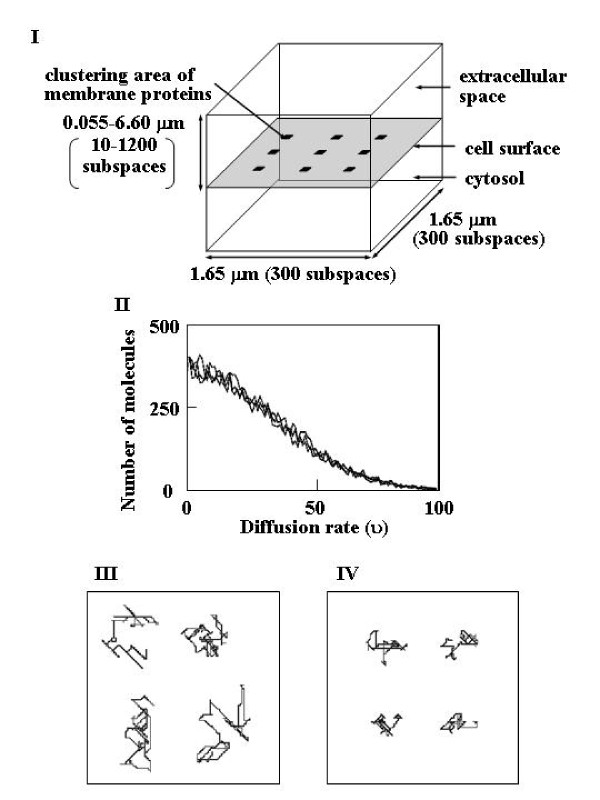
**Cell model for simulation, molecular diffusion rates, and receptor movement**. (A) The cell model used for simulation. See text for details. (B) Distribution of diffusion rates (υ). For details, see text. (C,D) Movement of R was plotted for 1 × 10^4 ^steps at intervals of 10 steps. The diffusion rates of R used were υ(C) and 0.1υ(D). The numbers of subspaces were 600 × 600 × 1 (C) and 300 × 300 × 1 (D).

In the present study, we assumed a simplified model in which the cell surface is represented as a 2-dimensional plane between 3-dimensional extracellular and cytosolic spaces (Figure 1-I). The cell surface and the extracellular space were divided into subspaces. Real-type pseudo uniform random numbers (N) with the range 0 ≤ N < 1 were generated as reported previously [15]. Each molecule was assumed to undergo random motion with a diffusion rate (υ) that has a pseudo-normal probability distribution from 0 to 100. υ was generated as described in Table 1, and the resulting distribution is shown in Figure 1-II. Each molecule has υ and its direction of movement (positive or negative direction on each axis), and molecules move into their neighboring subspace when τ < υ, where τ is a pseudo uniform random number (0 ≤ τ < 100) obtained as described in Table 1. υ was defined as the diffusion rate of the extracellular ligand and cytosolic proteins, and 0.1υ was used for the rate of membrane proteins and their complexes with the ligand. When υ = 0, the molecules remained in the same subspace. The diffusion rates and directions were updated for 1% of all molecules at each step, and this sample population was selected randomly. The trajectories of the receptors are shown in Figure 1-III and 1-IV. We assumed periodic boundary conditions; i.e., a molecule moved to the opposite side when it reached the boundary of its simulation box, except that when it reached the cell surface or its opposite boundary, it was reflected in the mirror direction.

**Table 1:** A list of integral random numbers

Used for	Range	Equations
Diffusion rate (υ)	0–99	Absolute value of (S/6-200), where $\text{S}=\sum_{\text{i}=1}^{12}\left({\text{N}}_{\text{i}}\times 200\right)$^(1)^
Selection of moving step (τ)	0–99	N × 100^(2)^

A pseudo uniform random number (N) from 0 to (1 - 1 × 10^-15^) was generated as described in the Methods.

^(1)^S is the integer of the obtained value, and S values of 100 or less were used.

^(2)^The integer of the obtained value was used.

Nine clustering areas were assumed for membrane components, as illustrated in Figure 1-I, and each area consisted of 3 × 3 subspaces with energy barriers at each boundary. The energy required to escape from the clustering area was defined as 23.7 kilojoules·mole^-1^. This means that the probability of escaping from the clustering area beyond its boundary was 0.01% at a temperature of 310 K. To avoid bias, the candidate for each trial was selected randomly. The molecules that were not selected were reflected in the mirror-direction at the boundaries of the clustering areas. Under these conditions, 99.2% of membrane components were clustered in the 9 clustering areas. When the escaping possibility was set to 0.1%, 92% of membrane components were clustered.

All molecules were initially distributed into randomly selected subspaces of their own compartments with equal probability, and 99.2% of the membrane proteins clustered within 1.2 × 10^6 ^steps under standard conditions.

The LR and LRA complexes were assumed to form in the membranes by the following reactions:

L + R ↔ LR, LR + A ↔ LRA

where L, R, and A are an extracellular ligand, a membrane receptor, and a membrane protein such as an adaptor or a linker, respectively. LR and LRA are binary and ternary complexes, respectively. Two molecules of different species may bind to each other when they occupy the same subspace. The binding probability and the dissociation probability of the complexes were defined as described previously [14].

LR was rapidly converted to LRA, since R and A clustered before L was added. Therefore, the rate constant of LRA formation (*k*) was calculated as follows:

d[LRA]/dt = *k*[L][R]

A cytosolic signal pathway was postulated as follows. The first cytosolic signal protein (B) is activated by the following reaction:

LRA + B → LRA·B → LRA·B* → LRA + B*,

where B* is the activated form of B. B* is inactivated by

I_B_* + B* → I_B_*·B* → I_B_*·B → I_B_* + B,

where I_B_* is the active form of enzyme I_B_, which inactivates B*.

The second signal protein (C) is activated by the following reaction:

B* + C → B*·C → B*·C* → B* + C*,

where C* is the activated C, and C* is inactivated by

I_C_* + C* → I_C_*·C* → I_C_*·C → I_C_* + C,

where I_C_* is the activated form of the enzyme that inactivates C*. We considered a signal pathway consisting of 5 signal proteins, B to F, all of which were subjected to the same reaction as described above. The probability of each reaction was defined based on its activation energy as described previously [14].

The source code of our simulation program was implemented with Visual Studio C++.net (Microsoft Co.), and the program was run on a personal computer with Windows XP or 2000 (Microsoft Co.).

## Results

### Validation of our simulation methods

In the previous simulation [14], the volume of a single subspace was defined as 1.728 (1.20^3^) nm^3^, but this volume is smaller than the size of a large number of protein complexes. Therefore, in this study we defined that a single subspace was a cubic box with a volume of 166.1 (5.497^3^) nm^3^, as described previously [16]. In this model, one molecule per subspace corresponds to a concentration of 10 mM. Each calculation step was assumed to take 0.02 milliseconds.

To validate our simulation procedure, the dissociation constant (Kd) of the following reaction in the cytosolic space was evaluated.

A + B ↔ AB

In the equilibrium state, the following equation holds:

P_1 _× N_A _× N_B_/N_S _= P_2 _× N_AB_,

where N_A_, N_B_, and N_AB _are the numbers of molecules A, B, and AB, respectively. P_1 _and P_2 _are the binding and dissociation probabilities and are defined as exp(-ΔE_1_/RT) and exp(-ΔE_2_/RT), respectively. Where E, R, and T are the activation energy, gas constant, and absolute temperature, respectively. N_S _is the number of subspaces in the cytosolic space. Since one molecule per subspace corresponds to a concentration of 10 mM as described above, the molarities of A (M_A_), B (M_B_), and AB (M_AB_) are given by N_A_/(100 × N_S_), N_B_/(100 × N_S_), and N_AB_/(100 × N_S_), respectively. By defining the dissociation constant (Kd) as (M_A _× M_B_)/M_AB_, we get Kd = P_2_/(P_1 _× 100).

The numbers of A and B were both set to 600, and the cytosolic space contained 300 × 300 × 100 subspaces. P_1 _was set to 0.670. As shown in Table 2, the simulated Kd values are in agreement with those estimated by P2/(P1 × 100).

**Table 2:** Kd at equilibrium

	Dissociation constant (Kd)		
P_2_			
	Theoretical [P_2_/(P_1 _× 100)]	Simulated^(1)^	Ratio^(2)^
3.35 × 10^-4^	5.00 × 10^-6^	4.94 × 10^-6 ^± 0.33 × 10^-6^	0.99
6.71 × 10^-5^	1.00 × 10^-6^	1.05 × 10^-6 ^± 0.06 × 10^-6^	1.05
3.36 × 10^-5^	5.02 × 10^-7^	5.12 × 10^-7 ^± 0.36 × 10^-7^	1.02
6.72 × 10^-6^	1.00 × 10^-7^	1.05 × 10^-7 ^± 0.06 × 10^-7^	1.05

		Mean	1.03

^(1)^mean value and standard deviation of (M_A_·M_B_)/M_AB _calculated from 5 simulations.

^(2)^simulated value/theoretical value.

To save calculation time, the definition of υ was changed from the previous definition [14]. Under the present definition, random movements were simulated (Figure 1-III and 1-IV). The diffusion coefficient (D) was calculated as follows:

D = [average value of {x(0)-x(t)}^2^+{y(0)-y(t)}^2^]/4t

for the receptor, and

D = [average value of {x(0)-x(t)}^2^+{y(0)-y(t)}^2^+ {z(0)-z(t)}^2^]/6t

for cytosolic proteins, where x(0), y(0), z(0), x(t), y(t), and z(t) are their positions in the x, y, and z directions at 0 and t seconds. The mean value for 1000 molecules was calculated. The cytosolic space and the cell surface contained 1200 × 1200 × 1200 and 1200 × 1200 × 1 subspaces, respectively. The average values were obtained from 4 sets of calculations, which took between 0.03 and 0.06 seconds. The diffusion coefficients of the cytosolic protein and the receptor were 10.8 ± 0.3 and 0.163 ± 0.008 (μm)^2^·second^-1^, respectively. These values reflect experimental data for membrane and cytosolic proteins previously reported in prokaryotes [17] and eukaryotes [18,19]. These data suggested that our simulation procedure is adequate to achieve our purpose. We assumed that the diffusion rate of the ligand is close to that of cytosolic molecules because the extracellular space and cytosol both contain a large number of molecules including proteins.

### Ligand-receptor membrane complex formation

It is generally accepted that receptors and associated membrane proteins such as adaptors and linkers are clustered in the micro-domains of the cell surface and that this clustering plays a role in intracellular signaling. Lipid rafts consisting of glycosphingolipids, cholesterol, and membrane proteins have been observed on the cell surface [20]. Other interactions of membrane proteins have been reported to involve the F-actin skeleton [21]. Previous simulations by our group suggested that the clustering of membrane proteins such as receptors and adapters before ligand binding to receptors stimulated cell activation [14], in agreement with previous experimental data showing that receptors are associated with other membrane proteins in non-stimulated cells [22-25]. Therefore, we postulated that the membrane proteins associated with signal pathways cluster before ligand binding.

**Figure 2:**
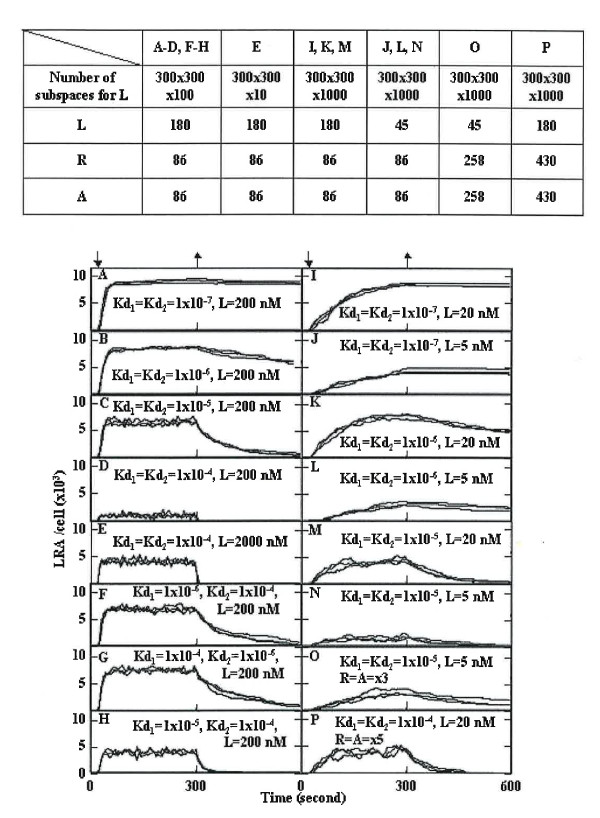
**Simulation of LRA formation**. The number of subspaces for L and the number of molecules were set as shown in the upper Table. The dissociation constants of LR (Kd_1_) and LRA (Kd_2_) are indicated in the Figure. The probabilities of LR and LRA formations were set to 0.67. L was added immediately before the 1.2 × 10^6^th step (down arrow, 24 seconds). After the 1.5 × 10^7^th step (upper arrow, 300 seconds), L was allowed to pass through the borders of the simulation box for L, except for the cell surface, and L was deleted in the outside area. Three simulation results are represented.

In the first simulation (model 1), the extracellular space and the cell surface consisted of 300 × 300 × 100 and 300 × 300 × 1 subspaces, respectively. The numbers of L, R, and A at zero time were set as described in Figure 2. The setting of L to 180 corresponds to 200 nM. The setting of R and A to 86 corresponds to approximately 10,000 molecules per cell, since the surface area of a spherical cell with a diameter of 10 μm is 314 μm^2^. More than 99% of R and A were clustered within 1.2 × 10^6 ^steps, and hence L was added immediately before the 1.2 × 10^6^th step. The concentration of extracellular ligands may not decrease with their binding to membrane receptors in situ because of the continuous supply of ligands from producing cells. To maintain a constant concentration of L, one molecule of L was set at the opposite side when L was bound to R on the cell surface. When LR dissociated, L was deleted from the opposite boundary until extracellular L decreased to its original level. After the 1.5 × 10^7^th step (t = 3 × 10^2 ^seconds), L was allowed to pass through the borders of the simulation box, except for the cell surface and was deleted from the borders.

When dissociation constants of LR (Kd_1_) and LRA (Kd_2_) were equally set to 1 × 10^-7 ^M or 1 × 10^-6 ^M, respectively, L was bound to 80% of the receptors (8,000 receptors per cell, Figure 2A and 2B). Even when the ligand concentration decreased to 20 or 5 nM, L was bound to 8,000 and 4,000 receptors, respectively, although the rate of LRA formation was slow (Figure 2I to 2L). In contrast to complex formation, dissociation of the complex was very slow with these dissociation constants. When both Kd_1 _and Kd_2 _were 10^-5 ^M, L was bound to approximately 6,000 receptors in the presence of 200 nM L, and fast dissociation was shown (Figure 2C). Five thousand and 2,000 complexes were formed when the concentration of L was 20 and 5 nM, respectively (Figure 2M and 2N). When both Kd_1 _and Kd_2 _were 10^-4 ^M, the complex level was 1,000 and 4,000 per cell in the presence of 200 and 2000 nM L, respectively (Figure 2D and 2E). However, experimental studies showed that a ligand concentration of less than 100 nM activated cells. When the cells contained a 5 fold excess of R and A (50,000 per cell), L was bound to approximately 4,000 receptors even when the concentration of L was 20 nM, and very rapid dissociation was demonstrated (Figure 2P).

These results suggest that the excess amount of receptors and its associate membrane proteins are not required for cell activation when the Kd is low or the concentration of L is high. However, it was shown that the prompt activation of the intracellular signal in the presence of a low amount of ligands and its rapid cessation after ligand clearance require an excess amount of receptors and membrane proteins. Cell activation with a low amount of ligands is favorable in situ.

When Kd_1 _and Kd_2 _were set to10^-6 ^M and 10^-4 ^M, respectively (Figure 2F), the simulation results were similar to that of Figure 2C. The product of the dissociation constant (Kd_1 _× Kd_2_) was 10^-10 ^M in both cases. The same result was again obtained when Kd_1 _and Kd_2 _were set to10^-4 ^M and 10^-6 ^M, respectively (Figure 2G).

The rate constants of LRA formation shown in Figure 2C (Kd_1 _× Kd_2 _= 10^-10 ^M), M (Kd_1 _× Kd_2 _= 10^-10 ^M), and H (Kd_1 _× Kd_2 _= 10^-8 ^M) were calculated to be 2.7 × 10^5^, 3.2 × 10^5^, and 1.4 × 10^5 ^M^-1^·s^-1^, respectively. These values are higher than those reported by Andrews, et al. [26], similar to rates observed in PMA (4 beta-Phorbol 12-myristate 13-acetate) treated cells [27], and lower than values reported in other studies [28,29].

### Negative feedback regulation of cytosolic signal pathways

**Figure 3:**
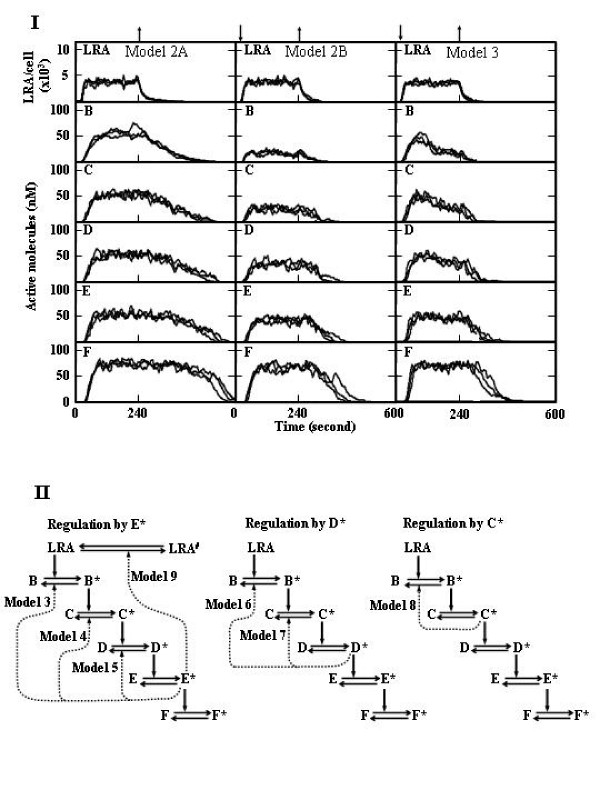
**Negative feedback regulation of the signal pathways**. I. The protein amounts and reaction probabilities were set as described in Additional file 1 and 2, respectively. L was added immediately before the 1.2 × 10^6^th step (down arrow, 24 seconds) and removed after the 1.2 × 10^7^th step (upper allow, 240 seconds), as described in the legend of Figure 2. Three simulation results are represented. II. Models for negative feedback regulation used in this study.

In the simulation of cytosolic signal transduction, the signal pathway was assumed to be as described in the Methods section. When Kd_1 _and Kd_2 _were set at 10^-5 ^M and 10^-4 ^M, respectively, ligands were bound to 40% of receptors in the presence of 200 nM L, and rapid formation and dissociation of LRA were demonstrated (Figure 2H). Therefore, we used these conditions for the signal transduction simulation. The concentrations of proteins and kinetic parameters were set as described in Additional file 1 and 2. All signal proteins (B to F) were activated rapidly after the ligand addition under conditions in which all signal proteins were activated at a high level (Figure 3-I, model 2A). However, inactivation of F was shown to be very slow under these conditions, even if the dissociation of the LRA complex was rapid (Figure 3-I, model 2A). As shown in Figure 3-I, when the levels of active B to D were low (Figure 3-I, model 2B), the activation rate of F (1.62 × 10^-9 ^± 0.08 M s^-1^, n = 3) was slower than the rate in model 2A (1.89 × 10^-9 ^± 0.03 M s^-1^, n = 3), but the inactivation of F was fast in model 2B.

Next, we postulated the following negative feedback regulatory mechanism (Figure 3-II) in which the amount of enzyme I_B_*, which inactivates B*, increased with the increase in E* as follows:

E* + I → E*·I → E*·I_B_* → E* + I_B_*,

where I is the precursor of I_B_*. Under this negative feedback regulation, the rapid activation of F (3.57 × 10^-9 ^± 0.13 M s^-1^, n = 3) and prompt shutdown of active F were demonstrated (Figure 3-I, model 3). These simulation results led us to conclude that high levels of active intermediate signal proteins promote the activation of the final step of the signal pathway, and low levels of the active proteins are required for prompt signal shutdown.

### The mechanism in which the first signal protein is regulated in a negative feedback manner is more effective

**Figure 4:**
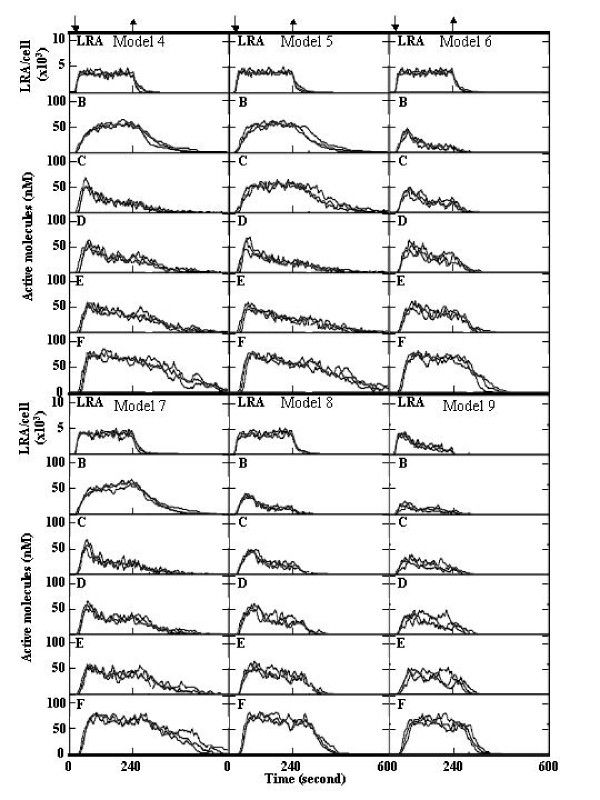
**Simulations of various types of negative feedback regulation**. The simulations were carried out as described in the legend of Figure 3. For detailed models, see the text. Three simulation results are represented.

Various models of negative feedback regulation were examined (Figure 3-II). When the level of B* was regulated by E*, the rapid activation of F and inactivation of F* were simulated (Figure 3-I, model 3), as compared with regulatory systems in which the level of C* (Figure 4, model 4) or D* (Figure 4, model 5) was regulated by E*, namely I_C_* and I_D_* increased with increases in E*. I_C_* and I_D_* were produced from I, as described above. Rapid shutdown was obtained when the level of B* was regulated by D* (Figure 4, model 6) or C* (Figure 4, model 8), as compared with model 7 (the level of C* was regulated by D*). These results suggest that negative feedback regulation of the B* level is the most effective for the prompt activation and cessation of signal pathways, but the regulation of B* by different molecules (C* to E*) had a similar effect on signal transduction.

It was shown that the membrane receptor complex was inactivated by the cytosolic signal protein in a negative feedback manner [30]. The final simulation was carried out under conditions in which LRA was inactivated by an increase in the level of E* (model 9) as follows:

$${\text{E}}^{*}+\text{LRA}\overset{\text{P}11}{\underset{\text{P}12}{⇄}}{\text{E}}^{*}\cdot \text{LRA}\overset{\text{P}13}{\underset{\text{P}14}{⇄}}{\text{E}}^{*}\cdot {\text{LRA}}^{\#}\overset{\text{P}15}{\to }{\text{E}}^{*}+{\text{LRA}}^{\#},$$

where LRA^# ^is unable to activate B. P11, P12, P13, P14, and P15 were set to 0.0200, 2.00 × 10^-6^, 1.99 × 10^-4^, 3.35 × 10^-7^, and 0.670, respectively. The results showed that the shutdown speed in model 9 was close to the speed in model 3.

## Discussion

The present simulation provided a kinetic explanation for why cells have a higher amount of receptors than is required to initiate signal transduction. When the dissociation constant is low, excess receptors are not required for cell activation, but signal shutdown is delayed after clearance of the ligand from the extracellular space. Even when the dissociation constant (Kd_1 _× Kd_2_) was 10^-8 ^M, the excess receptor was not required in the presence of 2 μM extracellular ligand, but such a high concentration of ligand may not represent physiological conditions. Therefore, an excess amount of receptors is useful for the rapid activation and inactivation of intracellular signal transduction when ligand concentrations are at a physiological level.

Our simulation results can be applicable to other models. For example, when ligand-receptor complexes without other membrane proteins initiate cellular signaling, the dissociation constant of LR more than 10^-9 ^M was essential for rapid signal cessation and an excess amount of receptors was required for activation at a ligand level below 1 μM (data not shown). If receptors are crosslinked by multivalent ligands, the following reactions are used

L + R ↔ LR and LR + R ↔ LRR instead of L + R ↔ LR and LR + A ↔ LRA.

Therefore, similar results could be obtained because both have kinetic similarity. The signal transduction that is deactivated by the binding of other membrane components to ligand-receptor complexes shows similar kinetics to the model 9 using the component instead of E* without P15.

Previous examinations of signal transfer pathways with the aid of computer simulations theoretically supported the negative feedback control of signal transfer observed experimentally [8-10], but its physiological meaning remained unclear. The present results clearly demonstrate that negative feedback regulation is required to promote the termination of a signal transfer system. The present simulation also suggests that negative feedback regulation of the first cytosolic signal protein is the most effective pathway. Experimental results have shown that the early steps of signal pathways are regulated in a negative feedback manner in many cases [31-36]. Other experimental studies revealed that the activation levels of signal proteins at the steady-state stage are very low [2-7], leading us to debate the physiological significance of such a low level of activation. The present simulation results have shown that a low activation level of signal proteins at the steady-state stage is of physiological importance for cellular signaling.

It has been proposed that negative feedback regulation represses fluctuations in signal transduction [10]. However, our present data revealed that the activation of signal proteins was not significantly stabilized by negative feedback regulation under our conditions (Figure 3 and 4).

Most computer simulations of biological phenomena have been performed with supercomputers using Unix based operating systems. This may be the reason why Monte Carlo techniques are still largely unavailable to most researchers. In contrast, our simulations were carried out on a conventional personal computer using the Windows XP or 2000 operating systems. The simulated values of diffusion coefficients and kinetic parameters were consistent with experimental data from literatures, demonstrating that our Monte Carlo simulation procedure is useful for kinetic analysis of cellular signal transduction. Furthermore, our simulation program can be used by other investigators for kinetic analysis of other biological phenomena with minor modifications, and neither special computing hardware nor special training is required.

## Conclusion

The present kinetic analysis revealed that excess receptors and negative feedback regulation promote activation and cessation of signal transduction when ligand concentrations are at a low physiological level.

## Competing interests

The authors declare that they have no competing interests.

## Authors' contributions

HK carried out the simulation and wrote the manuscript. RA and TY participated in the design of simulation program and preparation of the manuscript.

## Supplementary Material

Additional file 1 **Protein amounts**. Amounts of proteins used for simulation. Click here for file

Additional file 2 **Reaction probabilities**. Reaction probabilities of each steps used for simulation. Click here for file
