# Supplementary material for: Expression of excess receptors and negative feedback control of signal pathways are required for rapid activation and prompt cessation of signal transduction
Source: Cell Commun Signal. 2009 Mar 3;7:3. doi: 10.1186/1478-811X-7-3 (PMC2666736; doi:10.1186/1478-811X-7-3)
Supplement: Additional file 1 — Protein amounts. Amounts of proteins used for simulation. [file 1478-811X-7-3-S1.doc]

Additional file 1: Protein amounts

| Proteins | Model 2A | Model 2B | Model 3 | Model 4 | Model 5 | Model 6 | Model 7 | Model 8 | Model 9 |
| --- | --- | --- | --- | --- | --- | --- | --- | --- | --- |
| L | 200 | 200 | 200 | 200 | 200 | 200 | 200 | 200 | 200 |
| R | 10000 | 10000 | 10000 | 10000 | 10000 | 10000 | 10000 | 10000 | 10000 |
| A | 10000 | 10000 | 10000 | 10000 | 10000 | 10000 | 10000 | 10000 | 10000 |
| B | 100 | 100 | 100 | 100 | 100 | 100 | 100 | 100 | 100 |
| I_B_ | 20 | 100 | 0 | 20 | 20 | 0 | 20 | 0 | 100 |
| C | 100 | 100 | 100 | 100 | 100 | 100 | 100 | 100 | 100 |
| I_C_ | 50 | 50 | 50 | 0 | 50 | 50 | 0 | 50 | 50 |
| D | 100 | 100 | 100 | 100 | 100 | 100 | 100 | 100 | 100 |
| I_D_ | 50 | 50 | 50 | 50 | 0 | 50 | 50 | 50 | 50 |
| E | 100 | 100 | 100 | 100 | 100 | 100 | 100 | 100 | 100 |
| I_E_ | 50 | 50 | 50 | 50 | 50 | 50 | 50 | 50 | 50 |
| F | 100 | 100 | 100 | 100 | 100 | 100 | 100 | 100 | 100 |
| I_F_ | 50 | 50 | 50 | 50 | 50 | 50 | 50 | 50 | 50 |
| I | 0 | 0 | 200 | 500 | 500 | 500 | 500 | 500 | 0 |

units: molecules/cell for R and A, nM for others.
