# Supplementary material for: Expression of excess receptors and negative feedback control of signal pathways are required for rapid activation and prompt cessation of signal transduction
Source: Cell Commun Signal. 2009 Mar 3;7:3. doi: 10.1186/1478-811X-7-3 (PMC2666736; doi:10.1186/1478-811X-7-3)
Supplement: Additional file 2 — Reaction probabilities. Reaction probabilities of each steps used for simulation. [file 1478-811X-7-3-S2.doc]

**Additional file 2: Reaction probabilities**

| Molecules | P1 | P2 | P3 | P4 | P5 | P6 | P7 | P8 | P9 | P10 |
| --- | --- | --- | --- | --- | --- | --- | --- | --- | --- | --- |
| B | 0.670 | 3.35x10^-7^ | 0.100 | 3.35x10^-7^ | 0.670 | 0.202 | 2.03x10^-7^ | 1.99x10^-4^ | 3.35x10^-7^ | 0.670 |
| C | 0.670 | 3.35x10^-7^ | 0.100 | 3.35x10^-7^ | 0.670 | 0.670 | 6.74x10^-7^ | 0.600 | 6.69x10^-8^ | 0.670 |
| D | 0.670 | 3.35x10^-7^ | 0.100 | 3.35x10^-7^ | 0.670 | 0.670 | 6.69x10^-8^ | 0.600 | 6.69x10^-8^ | 0.670 |
| E | 0.670 | 3.35x10^-7^ | 0.100 | 3.35x10^-7^ | 0.670 | 0.670 | 6.69x10^-8^ | 0.600 | 6.69x10^-8^ | 0.670 |
| F | 0.670 | 3.35x10^-7^ | 0.600 | 3.35x10^-7^ | 0.670 | 0.202 | 2.03x10^-7^ | 2.00x10^-2^ | 3.35x10^-7^ | 0.670 |

The probabilities (P1 to P10) were defined as follows:

where S, K, and I are cytosolic signal proteins, activation enzymes, and inactivation enzymes, respectively. Molecules marked by an asterisk are the active form of each molecule.
